# Assessment of catalytic and antibacterial activity of biocompatible agar supported ZnS/CuFe_2_O_4_ magnetic nanotubes

**DOI:** 10.1038/s41598-022-08318-6

**Published:** 2022-03-16

**Authors:** Fereshte Hassanzadeh-Afruzi, Zeinab Amiri-Khamakani, Shahrzad Bahrami, Mohammad Reza Ahghari, Ali Maleki

**Affiliations:** Catalysts and Organic Synthesis Research Laboratory, Department of Chemistry, Iran University of Science and Technology, Tehran, 16846-13114 Iran

**Keywords:** Biochemistry, Catalysis, Green chemistry

## Abstract

The tubular magnetic agar supported ZnS/CuFe_2_O_4_ nanocomposite was fabricated via a simple procedure. Next, various properties of this nanocomposite were studied by employing multiple characterization techniques including FT-IR, EDX, SEM, TEM,VSM, XRD, and TGA. Then, the catalytic and antibacterial applications were evaluated for the fabricated nanocomposite. Based on the experimental result, the nanocomposite showed excellent catalytic activity to promote the multicomponent reaction between ethyl acetoacetate, hydrazine hydrate, aromatic aldehydes, and malononitrile to synthesize a variety of dihydropyrano[2,3-c]pyrazole derivatives with high yields (89–95%) in acceptable reaction times (20–40 min) under mild reaction conditions. It can be efficiently recycled and re-work in six consequent runs without notable reduction in catalytic productiveness. Furthermore, its antibacterial activity was assessed against *Staphylococcus aureus* (*S. aureus*) and *Escherichia coli* (*E. coli*) bacteria by the agar diffusion and plate-count methods. These results indicate that the width of the inhibition zone around the *S. aureus* (G^+^ bacterium) is more than that of *E. coli* (G^−^ bacterium). Moreover, the agar supported ZnS/CuFe_2_O_4_ nanocomposite exhibited strong prevention of the bacterial colonies’ growth.

## Introduction

The naturally originated based materials have been widely utilized in the fabrication of catalysts to design cleaner and environmentally friendly chemical processes. There exist several reports of using various polysaccharides such as cellulose^1,2^, chitosan^3–6^, alginic acid^7^, and newly agar^8,9^ as catalysts substrate for stabilization of inorganic nanoparticles. Using these macromolecules as support for upholding acidic or basic inorganic nanoparticles increases the catalytic productivity of the inorganic nanoparticles both by extending the catalytic surface and through lots of hydroxyl groups in the structure of these biopolymers. Therefore, in these hybrid composites, biopolymers and inorganic nanoparticles have a synergistic effect and amplify the catalytic activity of each other. Agar is extracted from red algae composed of a linear polysaccharide called agarose which mainly made of agar (~ 70%) and agaropectin^10^. The use of agar due to its fascinating properties such as biocompatibility, abundance, accessibility, and cheap has recently gained scientists' attention^11^. Another approach that has been frequently tried to increase the biocompatibility of these biopolymer-supported heterogeneous catalysts*,* is to give them magnetic properties. Magnetizing these heterogeneous catalysts makes it easier to separate them from the reaction media and effortlessly recycle them by a magnet bar. An important category of magnetic nanomaterials is spinel ferrites with the formula MFe_2_O_4_, where M represents a bivalent metal ion (M: Cu, Ni, Mn, Co, Mg, etc.)^11^. Owing to their specific electric and magnetic properties, they have found a broad range of industrial and medical applications for instance in drug delivery, magnetic resonance imaging (MRI), magnetic information storage devices, and sensors^7,12,13^. Among ferrites, copper ferrite plays a more prominent role in all these fields due to its unique Jahn–Teller effect of (Cu^2+^) ion^14,15^, moderate magnetization, and high coercivity^16^. In addition to the aforementioned applications, CuFe_2_O_4_ is also used as semiconductors^17^, catalyst^18,19^, photocatalyst^20^, and transition metal absorbent. Since antibacterial activity for both ZnS^21–23^ and CuFe_2_O_4_^16,24,25^ against gram negative and gram positive bacteria have been reported in previous articles, it is expected that a composite consisting of these two will have acceptable antibacterial activity. Compating infectious disease has been complicated by drug resistance. The increasing and indiscriminate use of antibiotics in recent decades had led to emerging the multidrug resistance in pathogenic and environmental microorganisms. As some pathogenic bacteria, such as vancomicine-resistance *E. coli and* meticilline *S. aureus* and, have become insensitive to existing antibiotics, alternative antibacterial materials need to be developed.

**Figure 1:**
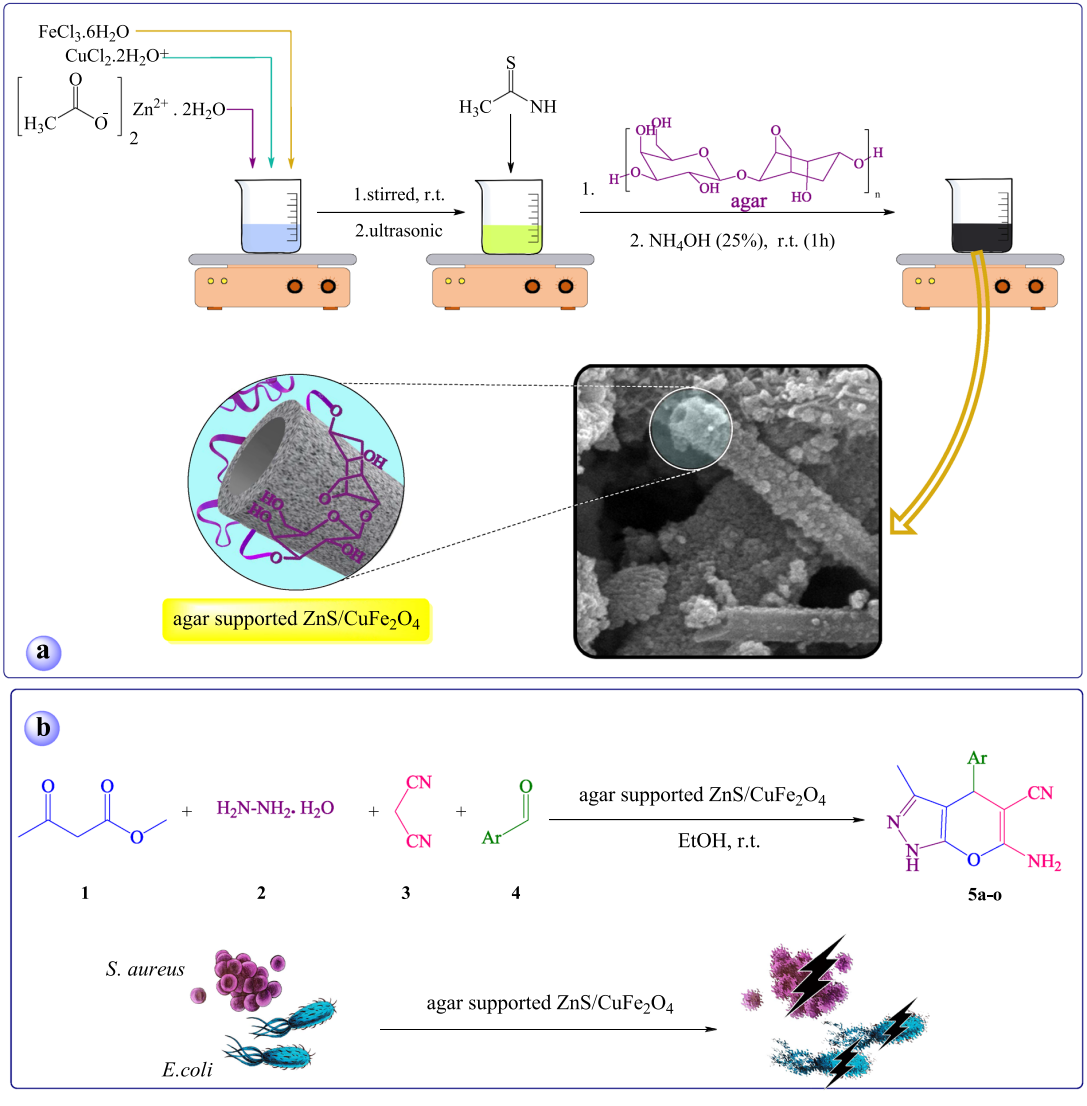
(**a**) The preparation route of the agar supported ZnS/CuFe_2_O_4_ and (**b**) its catalytic and antibacterial activities.

Dihydropyrano[2,3-c]pyrazole heterocyclic frameworks demonstrate various medicinal activities such as anti-inflammatory^26^, anticancer^27^, antimicrobial^28^, enzyme-inhibiting activity^29^, insecticidal^30^ and molluscicidal activity^31^. Therefore, because of the significant importance of these heterocycles in the matter of medicinal chemistry different methodologies and catalysts have been reported. Nevertheless, many of those procedures are productive and suggest benefit, still there is further demand for designing and developing more efficient and eco-friendly catalysts. Continuing our investigations on the hybrid nanocomposite, we prepared the agar supported ZnS/CuFe_2_O_4_ nanocomposite with tubular structure and evaluated its catalytic application in the one-pot four-component condensation reaction for the synthesis of pharmaceutically-active dihydropyrano[2,3-c]pyrazole derivatives and then its antibacterial activity against *S. aureus* and *E. coli* bacteria (Fig. 1).

## Experimental

### General

All chemical materials were bought from Merck, Sigma, and Aldrich. Thin-layer chromatography (TLC) was utilized to check the process of catalytic reactions. Mp (Melting point) of synthesized pyranopyrazole derivatives was checked with an Electrothermal 9100 apparatus. Energy-dispersive X-ray analysis of nanocomposite was carried out by using Numerix JEOL-JDX, 8030 instruments (20 mA, 30 kV). FT-IR spectra of samples were recorded on a Shimadzu IR‐470 spectrometer (using KBr pellets). The morphology and distribution of particle size of samples were studied by microscopic images obtained by SEM, VEGA2 TESCAN, and TEM, Philips CM200 instruments. XRD pattern of nanocomposite was recorded on Bruker D8 Advance X-ray diffractometer. ^13^C NMR and ^1^H NMR spectra of synthesized pyranopyrazole derivatives were taken using the Bruker DRX-500 Avance spectrometer at 125 MHz and 500 MHz, respectively. The magnetic behavior of the samples was assessed using a VSM of Meghnatis Kavir Kashan Co., and the TGA was employed to investigate the thermal stability of the fabricated samples by the BAHR-STA 504 instrument.

### The preparation of agar supported ZnS/CuFe_2_O_4_ nanocomposite

At first, an aqueous solution of FeCl_3_.6H_2_O (0.54 g in 20 mL of deionized water) was prepared. Next, 0.17 gCuCl_2_.2H_2_O (1 mmol) and 0.22 gZn (OAc)_2_.2H_2_O (1 mmol) were added to the above solution under stirring at room temperature to obtain a clear solution. Later, 0.12 g (1.6 mmol) thioacetamide was added to the stirring reaction mixture. After 20 min, agar (0.3 g) was added to the aforementioned mixture, and the reaction was allowed to proceed for another 30 min. Following that, 10 mL of NH_4_OH was added dropwise to the vigorously stirring reaction mixture, the reaction was then continued for 40 min. After ultrasonication of the mixture for 15 min, the black precipitate of the product was achieved then gathered with an external magnet and was washed with deionized water (5 × 20 mL) and acetone (2 × 10 mL). Finally, it was dried at room temperature.

### Typical procedure for the synthesis of dihydropyrano[2,3-c]pyrazole derivatives

For dihydropyranopyrazole synthesis, a mixture of 1.0 mmol ethyl acetoacetate, 1.2 mmol hydrazine hydrate, 1.0 mmol aldehyde, and 1.0 mmol malononitrile were and 0.02 g of the agar supported ZnS/CuFe_2_O_4_ catalyst in ~ 2 mL of EtOH in a round-bottom flask were stirred at room temperature for a required time until completion of the MCR and delivering the products. The progress of the reactions was monitored by TLC. After reactions were completed, hot EtOH was added to the reaction mixture to dissolve the product, then the undissolved magnetic catalyst was separated by using a magnetic and filtration. The pure dihydropyrano[2,3-c] pyrazole derivatives were gained by recrystallization of crude products from EtOH.

### Spectral data of the several products

6-Amino-3-methyl-4-(3-nitrophenyl)-1,4-dihydropyrano[2,3-c]pyrazole-5-carbonitrile (**5e**): ^1^H-NMR (DMSO‐d_6_): δ (ppm): 1.80 (s, 3H, Me), 4.88 (s, 1H, CH), 7.05 (s, 2H, NH_2_), 7.66 (m, 2H, H-Ar), 8.02 (s, 1H, H-Ar), 8.12 (d of d, J = 7.7 Hz, 1H, H-Ar), 12.20 (s, 1H, NH); ^13^C NMR (DMSO-d_6_); δ (ppm): 9.75, 35.66, 56.17, 96. 65, 120.49, 121.83,121.96, 130.22, 134.36, 135.89, 146.81, 147.88, 154.69, 161.13.

6-Amino-4-(4-chlorophenyl)-3-methyl-2,4-dihydropyrano[2,3-c]pyrazole-5-carbonitrile (**5b**): ^1^H-NMR (DMSO-d6): δ (ppm): 1.79 (s, 3H, Me), 4.63 (s, 1H, CH), 6.91 (s, 2H, NH2), 7.19–7.20 (d, J = 8 Hz, 2H, H-Ar), 7.37–7.38 (d, J = 8 Hz, 2H, H-Ar), 12.12 (s, 1H, NH); ^13^C NMR (DMSO-d_6_); δ (ppm): 10.18, 36.04, 57.3, 97.66, 121.06, 128.91, 129.82, 131.69, 136.12, 143.95, 155.19, 161.38.

6-Amino-4-(4-hydroxyphenyl)-3-methyl-2,4-dihydropyrano[2,3-c]pyrazole-5-carbonitrile(**5j**): ^1^H-NMR (DMSO-d6); δ (ppm): 1.78 (s, 3H, Me), 4.47 (s, 1H, CH), 6.68–6.69 (d, J = 8 Hz, 2H, H-Ar), 6.76 (s, 2H, NH2), 6.94–6.96 (2H, d, J = 8 Hz, 2H, H-Ar), 9.25 (s, 1H, OH) 12.03 (s, 1H, NH); ^13^C NMR (DMSO-d6); δ (ppm): 10.2, 35.95, 58.29, 98.53, 115.57, 121.32, 128.88, 135.22, 135.96, 155.23, 156.48, 161.09.

### Antibacterial activity of the agar supported ZnS/CuFe_2_O_4_ nanocomposite

The antibacterial performance of the agar supported ZnS/CuFe_2_O_4_ nanocomposite was assessed against both the G^−^ bacterium (*E. coli*) and the G^+^ bacterium (*S. aureus*) using agar well disk diffusion and the colony counting methods. Before experimental tests, all instruments had been sterilized for about 15 min at 121 °C in an autoclave. The tested bacteria were swabbed onto Mueller–Hinton agar as a solid growth medium. Agar well disk diffusion tests were conducted by the addition of sample-loaded disk to Muller-Hinton agar plates containing 0.5 McFarland turbidity of *E. coli* bacterium and *S. aureus* bacteria. The prepared samples were placed in the incubator at 37 °C for 24 h. The width of inhibition zones around the discs was measured in millimeters to vet the antibacterial performance of the nanocomposite against the studied bacterial pathogens. For the colony counting method, *S. aureus* (ATCC 12600) and *E. coli* (ATCC 9637) McFarland turbidity standard was added to 0.2 mL DMSO and a 0.1 g of each sample, then they stirred for 1 h and then 0.01 mL of the solution was added to Muller-Hinton agar plates. After that dishes were incubated for a day at 37 °C for bacterial growth and colonies formation.

## Rusult and discussion

### Characterization

**Figure 2:**
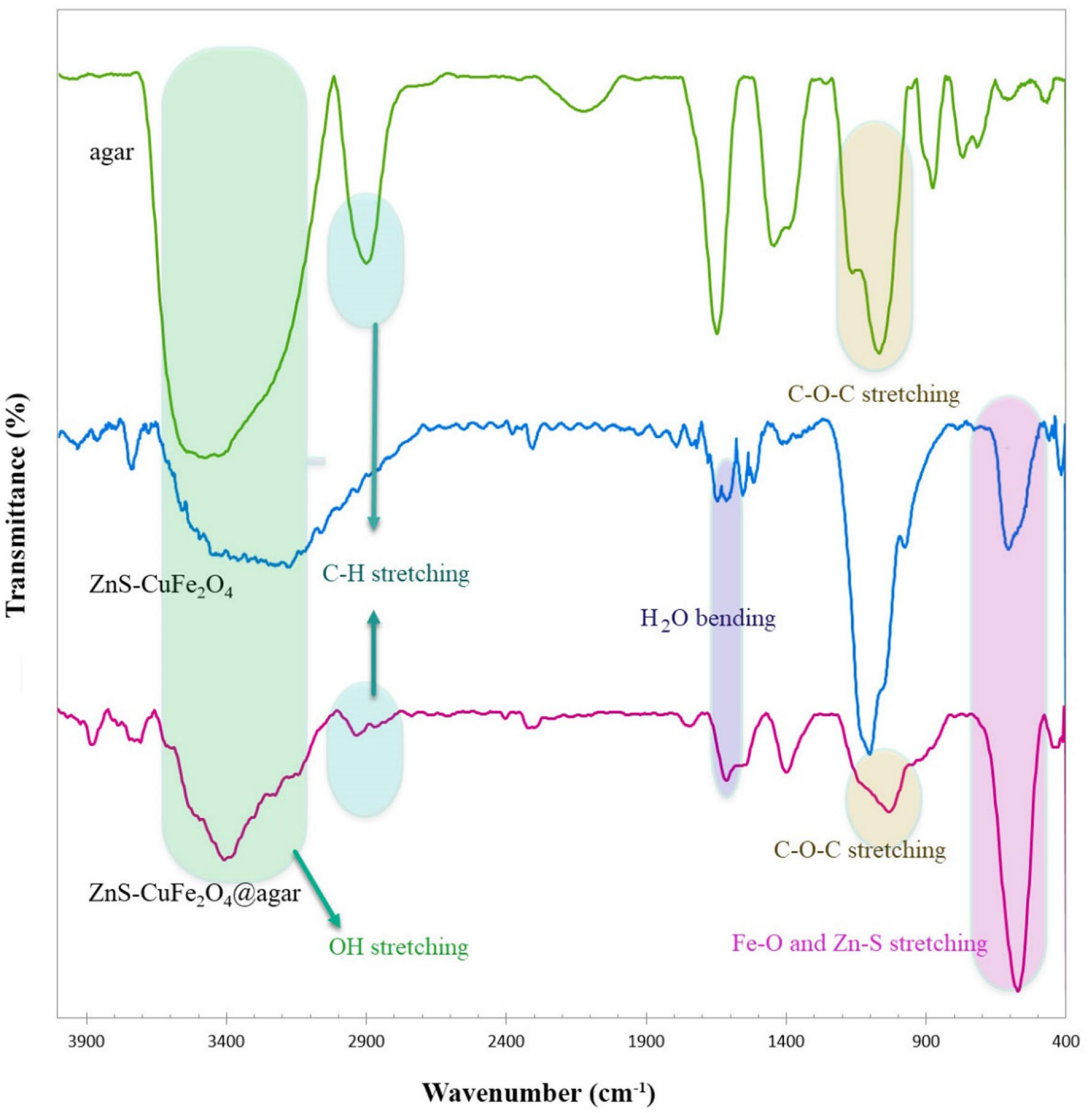
FT-IR spectra of the agar, ZnS/CuFe_2_O_4_ and the agar supported ZnS/CuFe_2_O_4_.

FT-IR (Fourier-transform infrared) spectra of agar and ZnS/CuFe_2_O_4_ hybrid and the agar supported ZnS/CuFe_2_O_4_ hybrid is presented in Fig. 2. Distinctive absorptions of agar in the spectrum appeared at 1050 (the glycosidic bond), 1157 (other C–O–C bonds), 2873 (aliphatic C‒H stretching vibration), 3400 cm^−1^ (O–H stretching vibration)^32^. The FT-IR spectrum of ZnS/CuFe_2_O_4_ hybrid (b) represents absorption at 603 cm^−1^ ascribed to the Fe^3+^‒O located at the tetrahedral sites^18,33,34^ which overlapped with Zn‒S stretching vibration^35,36^. Additionally, absorption at 1102 cm^−1^ is most likely related to the stretching vibration of S–O bond^37^. The absorption at 1633 cm^−1^ is attributed to the bending vibration of H_2_O^34,38,39^. The broadband at 3200–3600 cm^−1^ is ascribed to stretching vibration of O–H and adsorbed H_2_O on the surface of ZnS/CuFe_2_O_4_. The IR spectrum of agar supported ZnS/CuFe_2_O_4_ showed distinctive absorption including absorption band at 563 cm^−1^ which correspond to Fe^3+^‒O and Zn‒S of ZnS/CuFe_2_O_4_ hybrid_,_ absorption at 1015 cm^−1^ related to C–O stretching vibration, absorption at 2935 is assigned to aliphatic C-H, and a broad absorption centered at the wavelength of 3400 cm^−1^ comes from stretching vibration of hydroxyl group which abundant both in agar and the surface of ZnS/CuFe_2_O_4_ NPs. Absorption observed in the FTIR spectrum of the nanocomposite is in consistent with functional groups present in the structure of the nanocomposite and confirms the successful construction of the described nanocatalyst.

**Figure 3:**
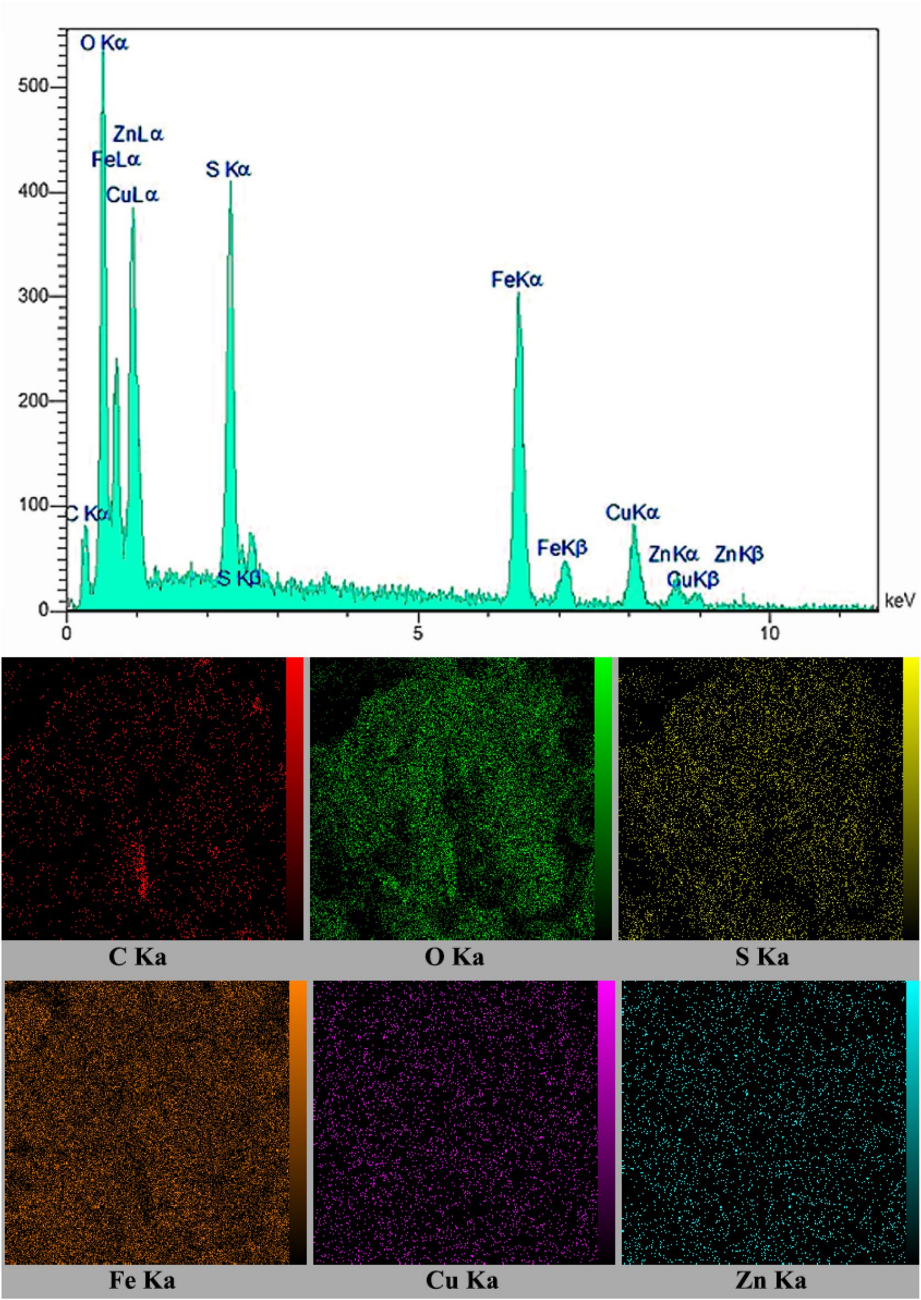
EDX spectra and elemental mapping image of of the agar supported ZnS/CuFe_2_O_4_.

To recognize the constituents elements of the agar supported ZnS/CuFe_2_O_4_, EDX (energy dispersive X-ray analysis) analysis was applied. The elemental analysis presented in Fig. 3 exhibited that the constituent elements in the nanocomposite are Fe, Cu, Zn, S, C, and O. Additionally, the distribution of elements in this agar supported ZnS/CuFe_2_O_4_ nanocomposite can be seen in the EDX mapping image.

**Figure 4:**
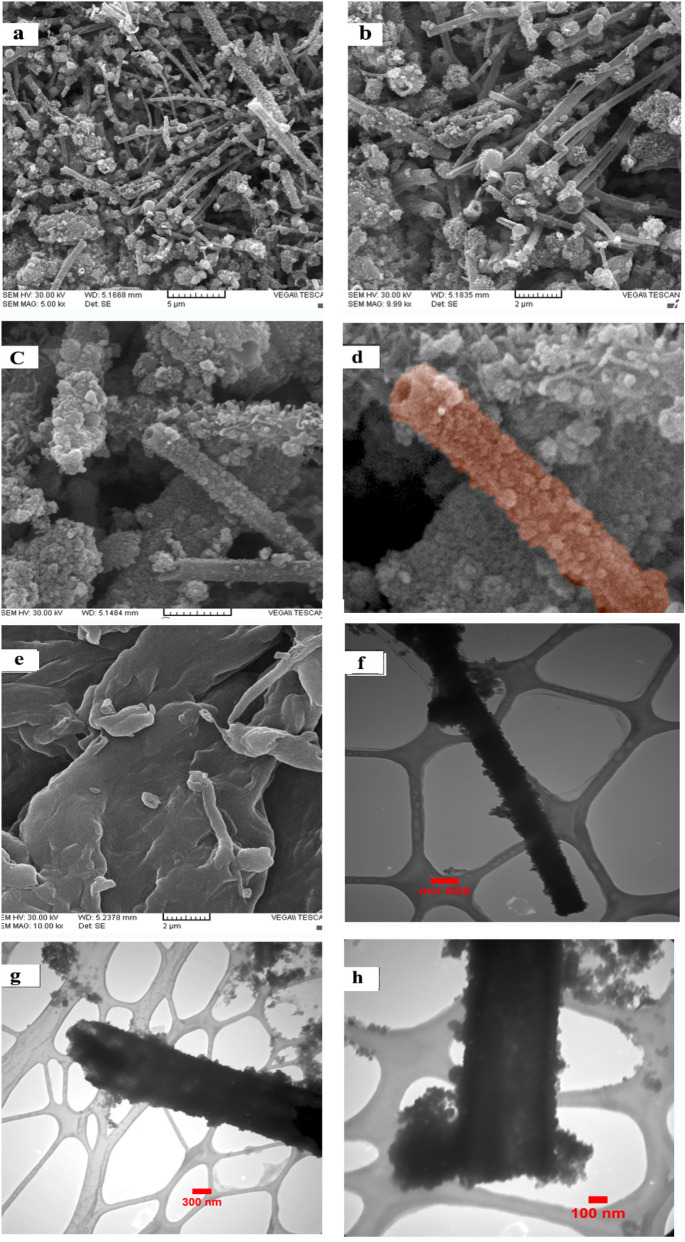
SEM images of (**a**–**d**) the agar supported ZnS/CuFe_2_O_4_, (**e**) agar, and (**f**–**h**) TEM images of the agar supported ZnS/CuFe_2_O_4_.

Scanning electron microscopy (SEM) and transmission electron microscopy (TEM) analyses were performed to study the morphology and particle size distribution of the the agar supported ZnS/CuFe_2_O_4_ nano biocomposite and neat agar as demonstrated in Fig. 4 in different magnifications. The SEMimages showed that agar supported ZnS/CuFe_2_O_4_ has tubular morphology. It can be deduced that agar not only contributes to the formation of these tubes as the catalyst substrate but also acts as the matrix of the organic–inorganic composite. The tubes of the agar supported ZnS/CuFe_2_O_4_ are distributed in the agar matrix and agar fibers covered the surface of tubes. The reason that led us to this interpretation is that SEMmicrographs of neat agar have also been taken and as it is observable in Fig. 4, agar doesn’t have a tubular, rod-like or cylindrical shape by itself. But the rod-like structure for both ZnS^40,41^ and CuFe_2_O_4_^33,42^ has been reported. So, with regard to proofs for composite comparing of ZnS and CuFe_2_O_4_, the formation of a tubular structure is not far from expectations. For a more detailed structural study of this catalyst, TEManalysis was performed. TEMimages of the agar supported ZnS/CuFe_2_O_4_ nanocomposite in Fig. 4 completely confirm the structure that was specified via the SEM images. Cylinder-shaped objects with a diameter of approximately 190 and 70 nm in different magnifications are observable in these images.

**Figure 5:**
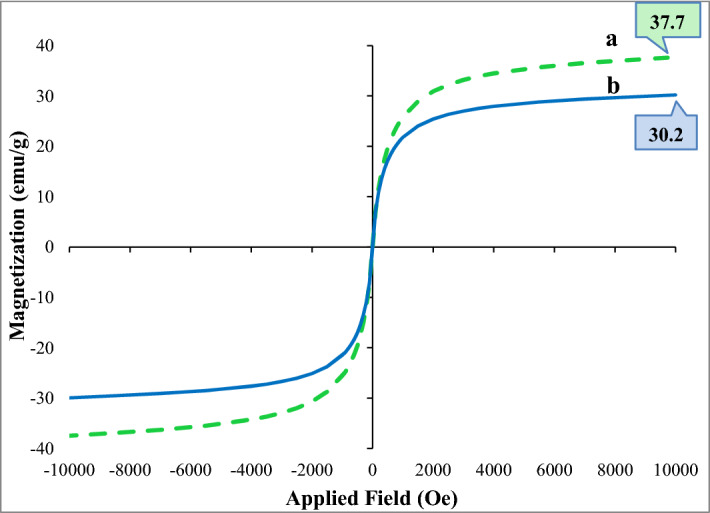
Magnetic-hysteresis curves of (**a**) ZnS–CuFe_2_O_4_ and (**b**) agar supported ZnS/CuFe_2_O_4_.

The inherent magnetic properties of agar supported ZnS/CuFe_2_O_4_ hybrid catalyst and ZnS/CuFe_2_O_4_ were measured by VSM machine. As is observable in Fig. 5, no hysteresis loop has appeared in the S-like magnetization curve which revealed that both coercivity (Hc) and remanence (Mr) is zero which confirmed the superparamagnetic nature of both examined samples. The room temperature magnetic measurement from − 10,000 to + 10,000 oersted showed that the saturation magnetization (Ms) value of agar supported ZnS/CuFe_2_O_4_ in comparison with that of the ZnS/CuFe_2_O_4_ hybrid has decreased to 30.2 emu g^−1^, which is attributed to the addition of agar to the catalyst composition that has reduced the mass percentage of magnetic ZnS/CuFe_2_O_4_. Despite this decrease in Ms, the hybrid catalyst still retains a high magnetic property for easy separation by a magnet from the reaction mixture.

**Figure 6:**
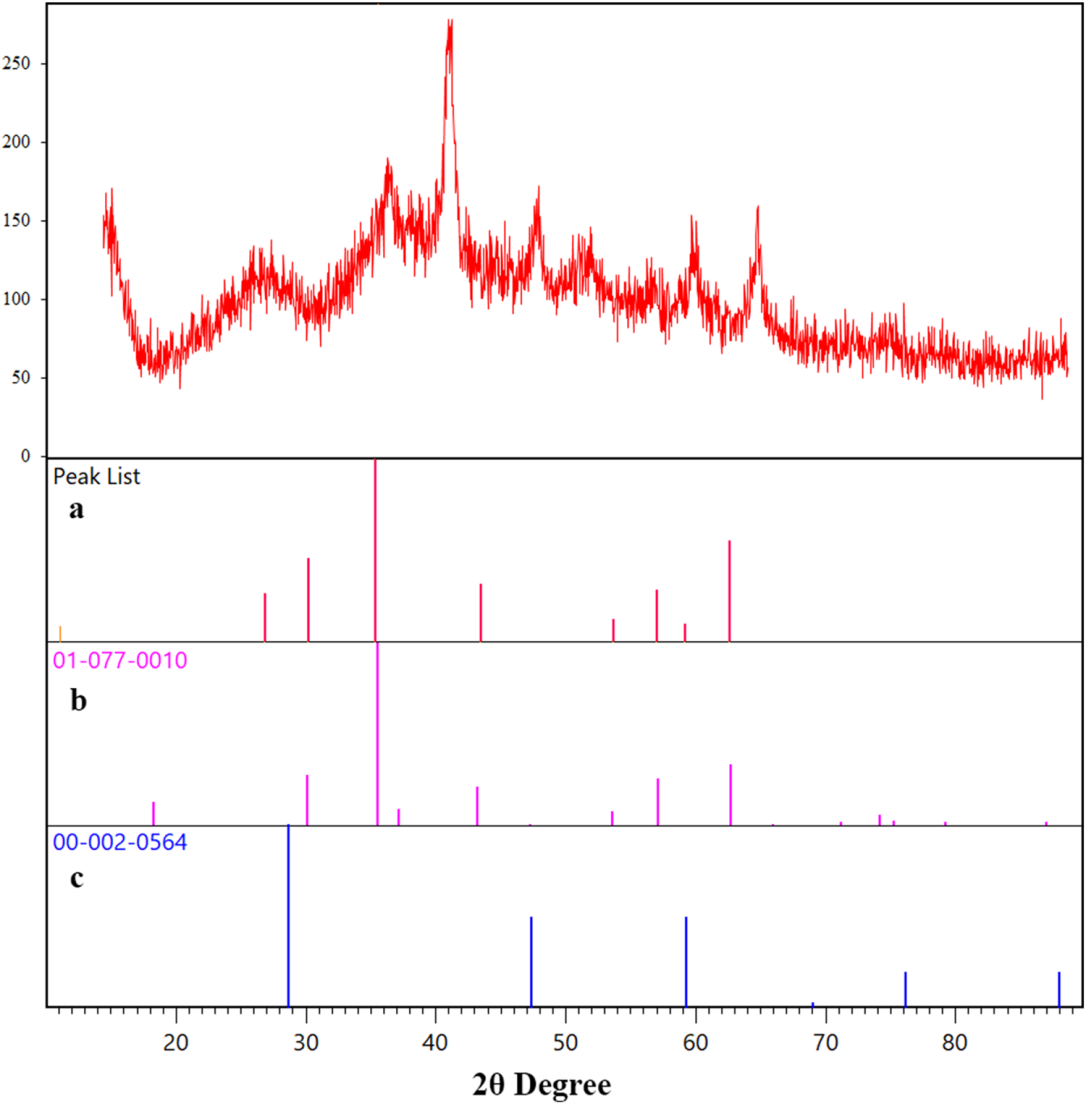
The XRD pattern of (**a**) the agar supported ZnS/CuFe_2_O_4_ (**b**) standard CuFe_2_O_4_ and (**c**) standard ZnS.

The X-ray diffraction pattern of the agar supported ZnS/CuFe_2_O_4_ in comparison with that of the reference database patterns of CuFe_2_O_4_ and ZnS was studied and the resulting diffractogramare illustrated in Fig. 6. Nine diffraction peaks was observed on the XRD pattern of this nanocatalyst at ~ 18.3°, 30.4°, 35.8°, 43.5°, 47.4°, 53.7°, 57°, 62.70°, 73.2°. Diffraction at 18. 32°, 30.55°, 35. 86°, 43.76°, 53.92°, 57.03°, 62.15°, 73.18° can be indexed to (1 0 1), (2 0 0), (2 1 1), (2 2 0), (3 1 2), (3 0 3), (2 2 4) and (3 0 5) crystal plane of cubic CuFe_2_O_4_ with JCPDS card no. 00-034-0425. Three peaks at 2ϴ ~ 47.40, 57°, and 73.2° are related to (2 2 0), (3 1 1), (3 3 1) lattice plane of cubic zinc blend with JCPDS card no. 00-005-0566. The peak at ~ 73° attributing to (3 0 5) Miller indices of CuFe_2_O_4_ most likely has overlapped with (3 3 1) crystal plane of ZnS. More studies were conducted to ensure that nanocomposite had no impurities. It was found that diffractogram of the agar supported ZnS/CuFe_2_O_4_ nanocomposite did not exhibit any peaks related to probable impurities such as CuO at about 2ϴ = 38.5°^43,44^, Cu_2_O around 2ϴ = 42.6°^45^ and rhombohedral α-Fe_2_O_3_ about 2ϴ = 24–24.5° and 33.1–33.5°^42,44^. The average crystallite size in the agar supported ZnS/CuFe_2_O_4_ was determined at about 21 nm using the Scherer equation.

**Figure 7:**
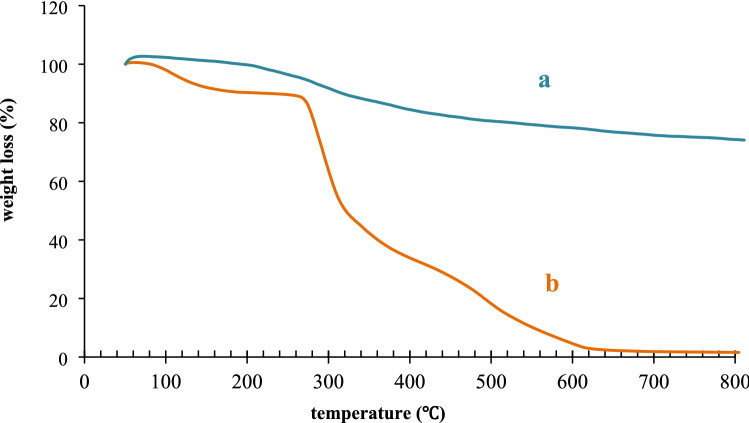
TGA of (**a**) the agar supported ZnS/CuFe_2_O_4_ and (**b**) agar.

The thermal stability of agar supported ZnS/CuFe_2_O_4_ was evaluated via performing TGA (thermogravimetric analysis) at 50 < T < 800 °C at air atmosphere and heating rate of 10 °C/min. The thermogram of agar and agar-supported nanocomposite are depicted in Fig. 7. Vetting of agars mass loss and this agar-based nanocomposite demonstrated that agar supported ZnS/CuFe_2_O_4_ has maintained 75% of its weight up to 800 °C while agar has entirely decomposed before reaching 600 °C and it confirmed the higher thermal stability of agar supported ZnS/CuFe_2_O_4_ than neat agar. Therefore it can be concluded that the combination of agar with the hybrid of ZnS–CuFe_2_O_4_ has considerably enhanced its thermal resistance. A minor weight loss (2%) below 220 °C on the TGA curve of agar (b), is ascribed to the desorption of adsorbed water. As the temperature increased (250–500 °C), the descending slope of the mass loss thermograph becomes steeper*.* The mass loss at this temperature range is most likely due to the dehydroxylation as well as breaking of glycosidic bonds in agar chains to lower weight units, and the breaking of hydrogen bonds between polysaccharide chains and ZnS–CuFe_2_O_4_ hybrid. As the temperature increased, the agar decomposed further into its monomeric units. At temperatures above 500 °C, carbonation occurred and the remaining organic parts of the agar were converted to soot. Comparison between nanocomposite and agar thermograms showed that while the agar completely disintegrated at 600 °C, the nanocamyosite retained about 75% of its weight. therefore agar contained about 25% (w/w) of the agar supported ZnS/CuFe_2_O_4_ composite. According to the reported information on the thermal behavior of ZnS and CuFe_2_O_4_, the ZnS was thermally stable up to 500° C and a slight weight loss after this temperature was attributed to the conversion of ZnS to ZnO^46^, and the TGA CuFe_2_O_4_ curve, no significant weight loss was observed except the fairly severe weight loss before 300 °C, which was due to the loss of absorbed water^44^. Thus, it can be concluded that the mass loss of agar supported ZnS/CuFe_2_O_4_ continued from 520 till 640 °C is ascribed to the oxidation of ZnS to ZnO.

### Application of the agar supported ZnS/CuFe_2_O_4_ hybrid catalyst in the synthesis of dihydropyrano[2,3-c]pyrazoles

The catalytic application of agar supported ZnS/CuFe_2_O_4_ nano biocomposite was considered in the one-pot synthesis of dihydropyrano[2,3-c]pyrazoles derivatives. To optimize the synthesis condition of these heterocycles, the reaction of ethyl acetoacetate, hydrazine hydrate, malononitrile, and 3-nitrobenzaldehyde in 2 mL of EtOH and at ambient temperature was chosed as a model reaction. Firstly, the effect of catalyst loading on the speed of reaction and productivity was tested. It was found that without any catalyst and solvent the reaction did not progress well (Table 1, Entry 1). Running the reaction in presence of a solvent, once water, and in another test EtOH but again in the absence of the catalyst had almost the same results as entry 1(Table 1, Entry 2&3). While the addition of a little amount of the catalyst in the presence of EtOH caused rapid progress of the reaction rate and increased the yield of the reaction significantly (Table 1, Entry 4). According to the results of entries 4–7, the optimum amount of nanocatalyst for effective promoting of dihydropyrano[2,3-c]pyrazoles synthesis is 0.02 g. Using lower amounts of the catalyst leads to lower yields of the products and in the presence of a higher amount of nanocatalyst, no increase in the reaction productivity was observed. The model reaction has also been run in the presence of an optimum amount of the catalyst, but this time using water as the solvent (Entry 8) and the yield was lower than the yield of the same reaction in the presence of EtOH. Moreover, efficiency of agar supported ZnS/CuFe_2_O_4_ catalyst in the synthesis of dihydropyrano[2,3-c]pyrazole derivatives has been compared with the yield of the same reaction catalyzed by CuFe_2_O_4,_ ZnS/CuFe_2_O_4_, and agar under similar conditions in entry 9–12. As is observed in Table 2, during an identified time the catalytic efficiency of the agar supported ZnS/CuFe_2_O_4_ nanocomposite is higher than that of its constituent components.

**Table 1:** Optimizing the four component reaction conditions for the synthesis of dihydropyrano[2,3-c]pyrazole derivatives^a^.

Entry	Catalyst	Solvent	Catalyst loading (g)	Yield^b^ (%)
1	–	–	–	Trace
2	–	H_2_O	–	< 30
3	–	EtOH	–	< 30
4	Agar supported ZnS/CuFe_2_O_4_	EtOH	0.01	68
5	Agar supported ZnS/CuFe_2_O_4_	EtOH	0.015	78
**6**	**Agar supported ZnS/CuFe**_**2**_**O**_**4**_	**EtOH**	**0.02**	95
7	Agar supported ZnS/CuFe_2_O_4_	EtOH	0.03	95
8	Agar supported ZnS/CuFe_2_O_4_	H_2_O	0.02	80
9	CuFe_2_O_4_	EtOH	0.02	50
10	ZnS/CuFe_2_O_4_	EtOH	0.02	65
11	Agar	EtOH	0.02	40

^a^Reaction conditions: 1 mmol ethyl acetoacetate, 1.2 mmol hydrazine hydrate, 1 mmol 3-nitrobezaldehyde and 1 mmol malononitrile, catalyst (10–20 mg).

^b^The yields relate to the isolated product, at r.t.

Significant values are in bold.

**Table 2:** The synthesis of different dihydropyrano[2,3-c]pyrazole derivatives under optimized conditions by utilizing agar supported ZnS/CuFe_2_O_4_ catalyst.


Entry	R1	Product	Time (min)	Yield^a^ (%)	Mp (°C)	
					Observed	Literature
1	2-Cl	**5a**	20	91	239–240	[241–244]^47^
2	4-Cl	**5b**	20	93	230–232	[232–233]^48^
3	2,4-Cl_2_	**5c**	25	92	196–198	[198–199]^49^
4	2-NO_2_	**5d**	25	91	225–228	[227–228]^49^
5	3-NO_2_	**5e**	20	95	196–198	[195–196]^50^
6	4-Me	**5f**	20	93	202–204	[204–206]^47^
7	4-OMe	**5g**	25	92	208–210	[210–212]^48^
8	3,4,5-(OMe)_3_	**5h**	40	89	208–211	[210–212]^48^
9	3-OH	**5i**	30	90	255–257	[253–256]^51^
10	4-OH	**5j**	30	90	221–223	[223–225]^48^
11	3,4-(OH)_2_	**5k**	40	89	175–177	[175–178]^52^
12	4-F	**5l**	20	94	203–205	[205–207]^52^
13	4-CH(Me)_2_	**5m**	20	92	213–215	[211–213]^49^
14	4-Br	**5n**	25	90	239–242	[242–246]^51^
15	2-OH-5-Br	**5o**	30	90	226–228	[226–227]^48^

^a^The yields relate to the isolated product.

The generality and repeatability of the present approach were evaluated by utilizing diverse aromatic aldehydes with electron releasing groups electron-withdrawing or substituents in the the synthesis of different dihydropyrano[2,3-c]pyrazoles. All tested benzaldehydes resulted in high productivity(89–95%) after suitable reaction time under mild reaction conditions.

To estimate the advantage of this catalyst compared to other previously reported catalysts in the synthesis of **5e** derivative, a comparison has been made between their catalytic performance and the results are exhibited in Table 3. The presented information prove the superiority of the present methodology in terms of biocompatibility of the catalyst, use of environmentally friendly solvent, obtaining the desirable products with high yields in a suitable time under mild reaction condition.

**Table 3:** Comparison of the catalytic performance of the the agar supported ZnS/CuFe_2_O_4_ catalyst with some other reported catalysts for the synthesis of dihydropyrano[2,3-c]pyrazole (product **5b**).

Entry	Catalyst	Conditions	Catalyst loading	Yield (%)	References
1	Isonicotinic acid	Solvent free, 85 °C	10 mol%	92	^53^
2	Tris-hydroxymethylaminomethane	H_2_O:EtOH (1:1), r.t	30 mol%	90	^54^
3	Lemon peel powder	EtOH, reflux	10 wt%	74	^55^
4	OPC-SO_3_H^a^	EtOH, 80 °C	0.02 g	89	^56^
5	TEA-Im-IL-Cu^b^	H_2_O, 80 °C	200 ml	85	^57^
6	PAN@melamine/Fe_3_O_4_	H_2_O/EtOH (1:1), 80 °C	0.01 g	95	^58^
**7**	**Agar supported ZnS/CuFe**_**2**_**O**_**4**_	**EtOH, r.t.**	**0.02 g**	**95**	**This work**

^a^Carbon powder derived from waste orange peel-SO_3_H.

^b^Triethanolamine–imidazole–ionic liquid–Cu.

Significant values are in bold.

### A plausible mechanism for the synthesis dihydropyrano [2, 3-c]pyrazole derivatives

**Figure 8:**
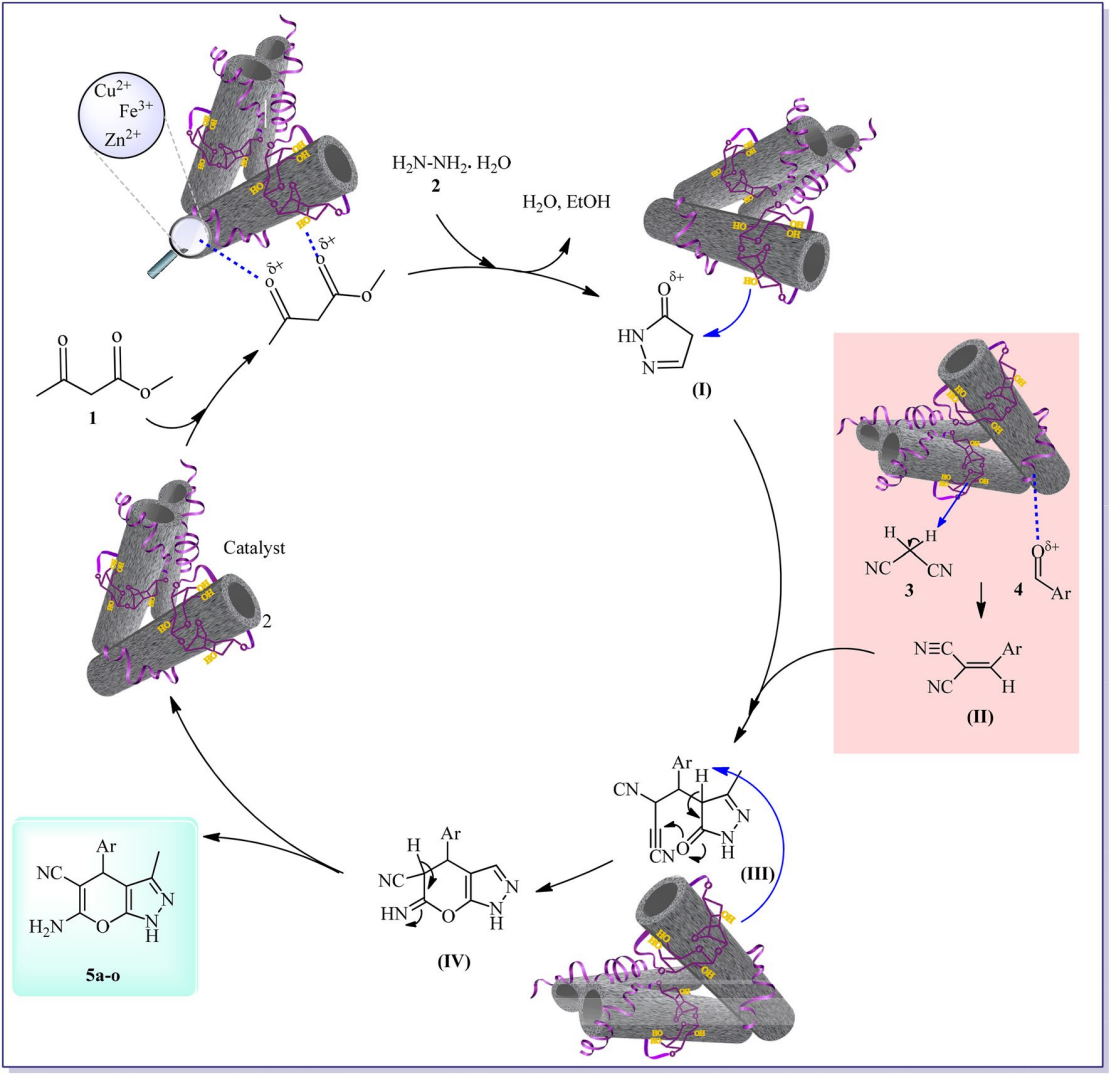
Proposed mechanism in the synthesis of dihydropyrano[2,3-c]pyrazole derivatives (**5a-o**) catalyzed by the agar supported ZnS/CuFe_2_O_4_ catalyst.

The agar supported ZnS/CuFe_2_O_4_ nanocomposite with different active sites: abundant hydroxyl in agar and Lewis acid site including Fe^3+^, Cu^2+^,Zn^2+^ in ZnS/CuFe_2_O_4_ hybrid play an influential role in conducting the all steps of this MCR as depicted in Fig. 8. Based on the reported information in the previous studies, the final product was obtained by several main steps^58,59^. At first, the agar supported ZnS/CuFe_2_O_4_ catalyst interacted with ethyl acetoacetate and activated their carbonyl groups through hydrogen bonding and the Lewis acid site, In the next step, the nucleophilic attack of hydrazine hydrate on activated ethyl acetate generated an intermediate **I** (pyrazolone ring) which was accompanied by the removal of ethanol and water molecules. On the other hand, the catalyst-activated malononitrile (by OH groups of catalyst) and activated aromatic aldehyde (by Lewis acid site and hydrogen bond) produced intermediate **II** (2-phenylidenemalononitrile) via the Knoevenagel reaction. Then, the catalyst-activated intermediates pyrazolone ring interact with 2-phenylidenemalononitrile via a Michael addition reaction to produce the intermediate **III**. Lastly, compound **IV** was formed by enolization and cyclization of intermediate **III** and dihydropyrano [2, 3-c]pyrazole derivatives (**5a-0**) were eventually produced by tautomerization of molecule **IV**.

### Catalyst recyclability

**Figure 9:**
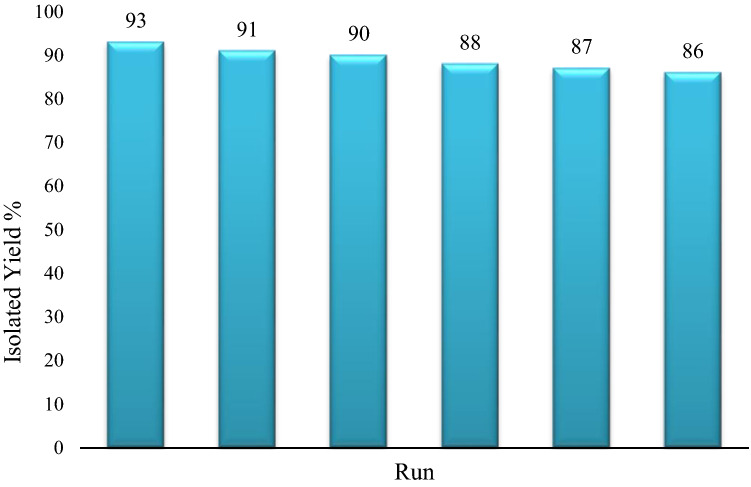
Recyclability of the agar supported ZnS/CuFe_2_O_4_ hybrid catalyst in the synthesis of **5b**.

The reusability of the agar supported ZnS/CuFe_2_O_4_ catalyst was evaluated in the synthesis of 5b. Because of the magnetic property of the catalyst, it can be effortlessly separated from the reaction mixture with the help of a magnet bar, washed repetitively with ethanol and distilled water and dried after each run. Fortunately, the catalyst deactivation was negligible after six successive runs in the synthesis of dihydropyrano[2,3-c]pyrazole (Fig. 9). The FT-IR spectrum of the recycled catalyst showed was almost identical to the fresh one (Fig. S1).

### Antibacterial activity of the agar supported ZnS/CuFe_2_O_4_ nanocomposite

#### Evaluation of agar diffusion

**Figure 10:**
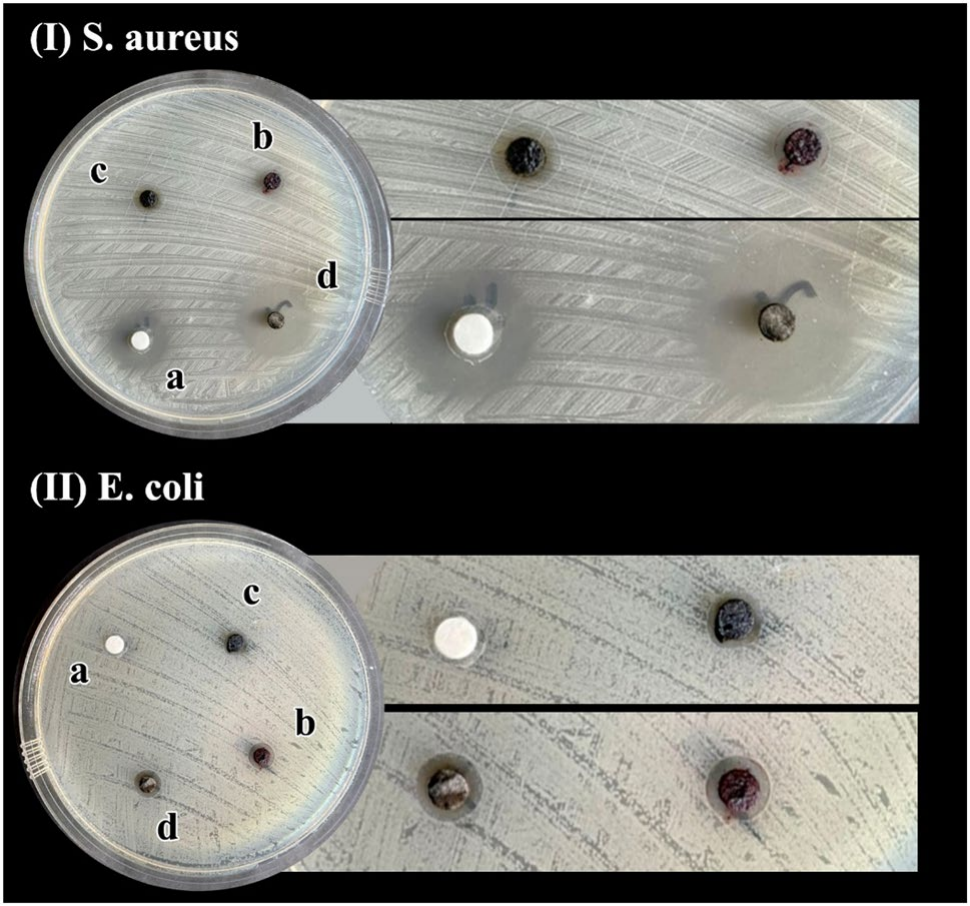
Inhibition zone of (**a**) ZnS, (**b**) CuFe_2_O_4_, (**c**) ZnS/CuFe_2_O_4_, and (**d**) the agar supported ZnS/CuFe_2_O_4_ against *E.coli* and *S. aureus* cultures.

To expand the application of the fabricated nanocomposite, the antibacterial efficiency of agar supported ZnS/CuFe_2_O_4_ nanocomposite, ZnS, CuFe_2_O_4_, and ZnS/CuFe_2_O_4_ against the *E. coli* (G^−^ bacterium) and *S. aureus* (*G*^+^ bacterium) by *in-vitro* study and agar diffusion method was examined. Comparative antibacterial performance of the agar supported ZnS/CuFe_2_O_4_, ZnS, CuFe_2_O_4_, and ZnS/CuFe_2_O_4_ against these two studied microorganisms were determined by measuring the zones of inhibition around each sample-loaded disc. The images of the zone of inhibition (ZOI) of all examined samples against *E. coli* and *S. aureus* can be observed in Fig. 10. Table 4 includes information about zone of inhibition width around discs. In general, what can be seen from these images is that the bacterial inhibition activity of the nanocomposite against *S. aureus* as a G^+^ bacterium is higher than that of *E. coli* as a G^−^ bacterium. But, the differences in the ZOI in *S. aureus* and *E. coli* strains can be ascribed to the differences in the membrane structure and composition of these bacteria. G^−^ bacteria has a complicated cell wall with internal and external membranes which composed of peptidoglycan, lipopolysaccharides, lipoproteins, and phospholipids molecules but the cell wall of G^+^ bacteria has a single membrane of peptidoglycan. Therefore, G^−^ bacteria with complex cell walls are more impressible for penetrating antibacterial agents. In addition, a comparison of the diameter of ZOI around all the samples studied for each plate shows that the antibacterial behavior of the agar supported ZnS/CuFe_2_O_4_ nanocomposite is largely due to the presence of ZnS and CuFe_2_O_4_ as well-known antibacterial species and also the synergistic effect between them.

**Table 4:** Width of ZOI for different prepared samples against *E.coli* and *S. aureus*.

Sample	ZOI (diameter), mm	ZOI (diameter), mm
	*S. aureus*	*E. coli*
ZnS	23.3	10.7
CuFe_2_O_4_	10.1	10.7
ZnS/CuFe_2_O_4_	13.2	9.9
Agar supported ZnS/CuFe_2_O_4_	29.7	11.7

Furthermore, the antibacterial activity of the agar supported ZnS/CuFe_2_O_4_ was compared with the relevant antibacterial materials reported in literature. Information about method of nanomaterials preparation, their shapes and the ZOI toward the target bacteria was summerizied in Table 5. As indicated in Table 5, the present nanocomposite exhibited comparable or even superior antibacterial effect than the reported material with greater zone of inhibiation.

**Table 5:** Example reports on the antibacterial activity of ZnS or CuFe_2_O_4_-contained materials.

Sample	Method	Shape	Bacteria	ZOI (mm)	References
ZnS	Chemical co-precipitation	Spherical	*P. aeruginosa*	5.0–14.0	^60^
			*Actinomycet*	5.0–16.0	
			*S. typhi*	12.0–23.0	
ZnS–cellulose nanocomposite	Chemical co-precipitation	Spherical	*E. coli*	12.0	^61^
ZnS nanomaterials	Chemical co-precipitation	Un-even grain	*E. coli*	9.0	^62^
ZnS	Chemical co-precipitation	Spherical	*S. aureus*	12.0	^63^
			*Bacillus*	14.0	
			*S. typhi*	12.0	
			*K. Pneumonia*	10.0	
			*Pseudomonas*	11.0	
ZnS with poly (vinyl acetate)	Electrospinning	Rice-grain shape	*E. coli*	9.0	^64^
			*S. aureus*	8.0	
Biotin capped Gd:ZnS	Microwave irradiation	Near spherical	*B. subtilis*	11.1–12.0	^65^
			*K. pneumonia*	13.0–21.2	
			*E. coli*	7.0–14.0	
			*P. syringae*	7.1–11.1	
Ce^3+^ doped CuFe_2_O_4_	Sol–gel	Spherical	*S. aureus*	14	^66^
			*K. pneumonia*	14	
CuFe_2_O_4_/Ti_3_C_2_ nanohybrids	Ultrasonication	–	*E.Coli*	23	^67^
			*S. aureus*	22	
			*K. pneumonia*	21	
			*P. aeruginosa*	22	
Agar supported ZnS/CuFe_2_O_4_	Chemical co-precipitation	Tublar	*E. coli*	11.7	This work
			*S. aureus*	29.7	

#### Plate-count method

**Figure 11:**
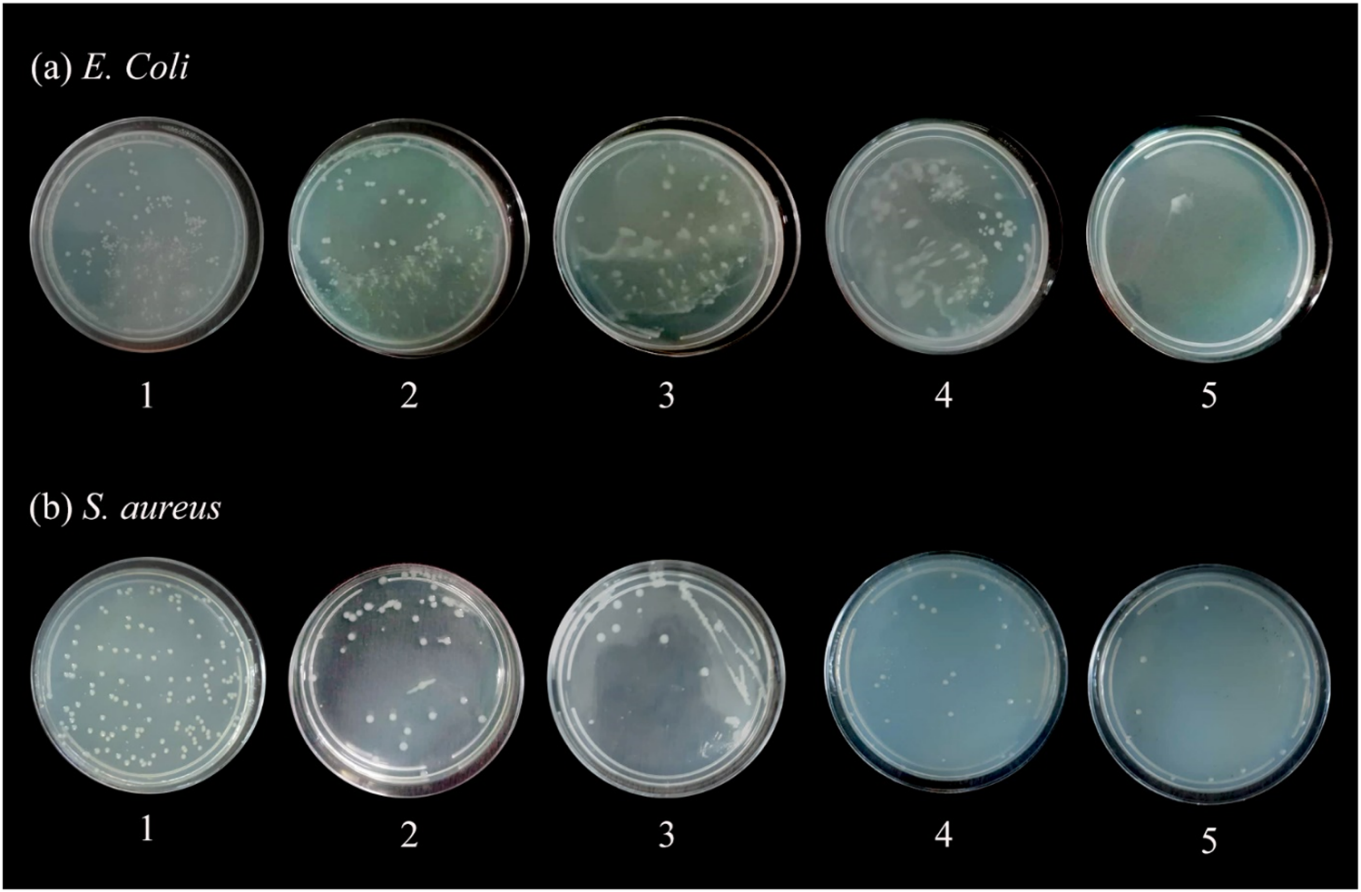
Colony counting images of *S. aureus* and *E. coli* after 24 h of incubation, (1) before treatment (control plate), after treatment with (2) agar, (3) ZnS, (4) CuFe_2_O_4_ and (5) agar supported ZnS/CuFe_2_O_4_.

The *S. aureus* and *E. coli* colonies after co-culture with the ZnS/CuFe_2_O_4_/agar and agar, ZnS and CuFe_2_O_4_ samples for 24 h were compared with the control sample. As shown in Fig. 11, the number of bacterial colonies was reduced considerably by treatment with the ZnS/CuFe_2_O_4_/agar nanocomposite in comparison with the control samples of *S. aureus* and *E. coli*. The ability of the ZnS/CuFe_2_O_4_/agar nanocomposite to decrease both bacterial colonies was much more than other studied samples including agar, ZnS and CuFe_2_O_4_. Howerver exact mechanism of antibacterial action of nanoparticles is still unclear, there are several mechanisms repoted in literature. The antibacterial effect of the agar supported ZnS/CuFe_2_O_4_ nanocomposite may be related to several resean include; (a) its effective interaction with the bacterial cell wall due to its high surface-to-volume ratio, (b) releasing the metal ions (Zn^2+^, Cu^2+^) and electrostatic interaction with bacteria cell walls with a negative charge, which had a destructive effect on the structures of bacteria cells membrane, irreversible DNA damage, and consequent bacterial cell death, and (c) their capability to produce reactive oxygen species (ROS) that damage the bacterial membrane , the extrusion of cytoplasm, disrupt cell function, and burst of bacteria^68^.

## Conclusions

The inorganic–organic agar supported ZnS/CuFe_2_O_4_ hybrid nanocomposite was fabricated via simple procedure using inexpensive easy accessible metal salts and agar as a biocompatible substrate. Next, the fabricated nanocomposite well characterized by different physicochemical analyses. Then, we investigated the catalytic and antibacterial performance of hybrid nanocomposites. The application of this catalyst in the tandem synthesis of biologically-active dihydropyrano[2,3-c]pyrazole derivatives was afforded the desired products with high yields (87–93%) under mild reaction conditions. Additionally, an examination of the antibacterial properties of this composite showed the high efficiency of agar supported ZnS/CuFe_2_O_4_ against *S. aureus* and *E. coli* microorgaism and was found to be eco-friendly with recoverability and durability*.* The result of SEM and TEM analyses showed that the agar supported ZnS/CuFe_2_O_4_ nanocomposite has a tubular shape. The TGA curve of agar supported ZnS/CuFe_2_O_4_ demonstrated its high thermal resistance so that, it retains about 75% of its mass until 800 °C which is attributed to the excellent thermals tability of inorganic parts of the nanocomposite. The VSM analyses displayed the paramagnetic behavior of prepared hybrid with Ms of around 30 emu/g which leads to its easy separation from reaction media, and reusability for at least six catalytic runs with negligible reduction in its catalytic efficiency.

## Supplementary Information

Supplementary Figures.
