# Supplementary material for: Assessment of catalytic and antibacterial activity of biocompatible agar supported ZnS/CuFe2O4 magnetic nanotubes
Source: Sci Rep. 2022 Mar 16;12:4503. doi: 10.1038/s41598-022-08318-6 (PMC8927411; doi:10.1038/s41598-022-08318-6)
Supplement: Supplementary file 1 — Supplementary Figures. [file 41598_2022_8318_MOESM1_ESM.pdf]

Supplementary Information

Assessment of catalytic and antibacterial activities of biocompatible agar supported ZnS/$CuFe_2O_4$ magnetic nanotubes

Fereshte Hassanzadeh-Afruzi, Zeinab Amiri-Khamakani, Shahrzad Bahrami, Mohammad Reza Ahghari and Ali Maleki*

Catalysts and Organic Synthesis Research Laboratory, Department of Chemistry, Iran University of Science and Technology, Tehran 16846-13114, Iran

**Corresponding author E-mail: maleki@iust.ac.ir; Fax: +98-21-73021584; Tel: +98-21-73228313*

Table of contents

Subject	*Page*

FT-IR spectrum of the recycled agar supported ZnS/$CuFe_2O_4$ catalyst (Fig.S1……………..…S2)

Copies of ^{1}H and ^{13}C NMR spectra of compounds **5b**, **5e**, **5j** (Figure S1-S6……………. S3-S8)

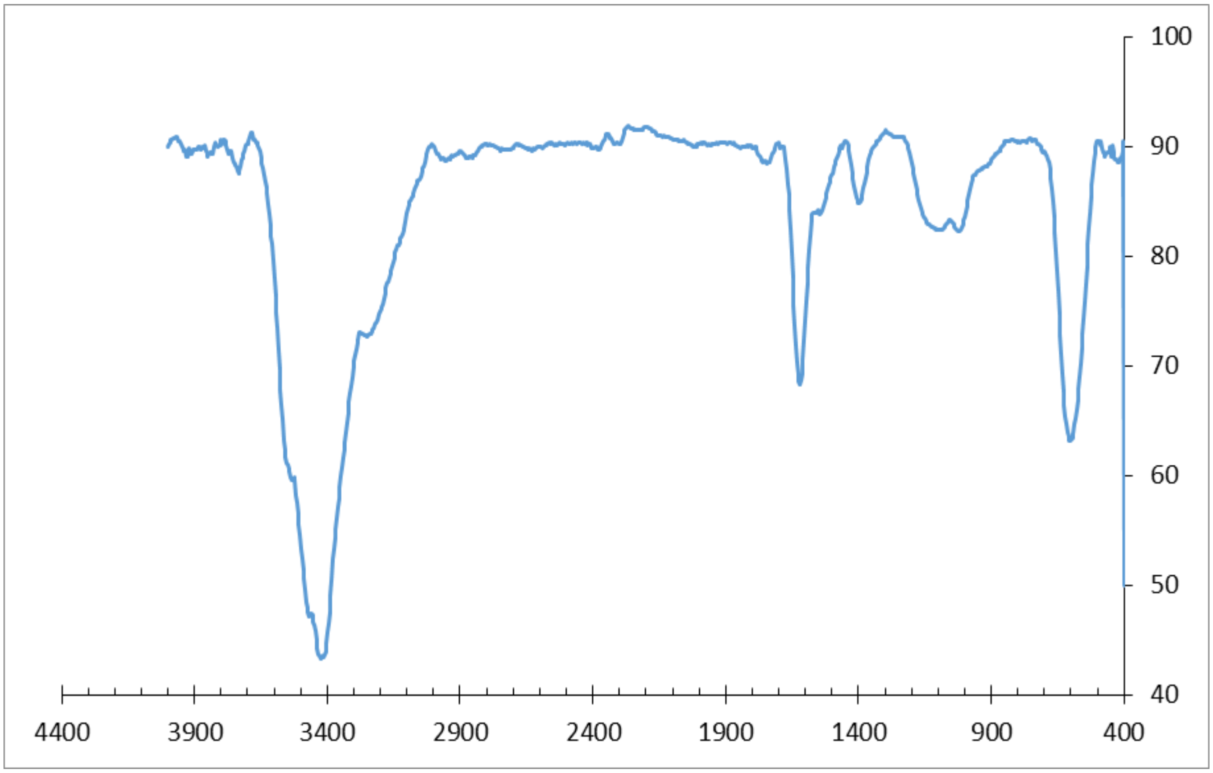

Fig. S1. FT-IR spectrum of the recycled $ZnS/CuFe_2O_4$/agar catalyst

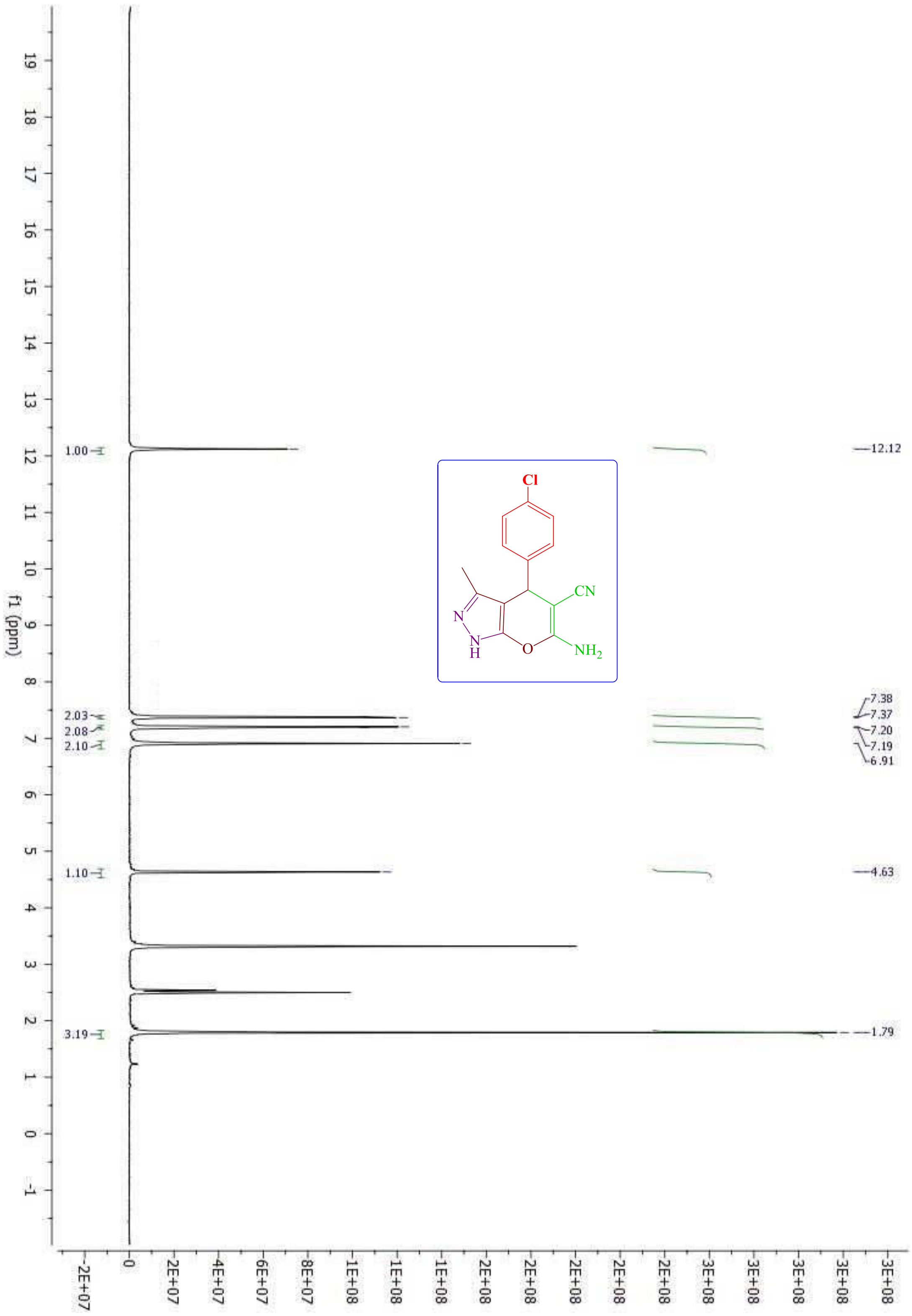

Figure S1. ^{13}H NMR spectrum of compound (**5b**)

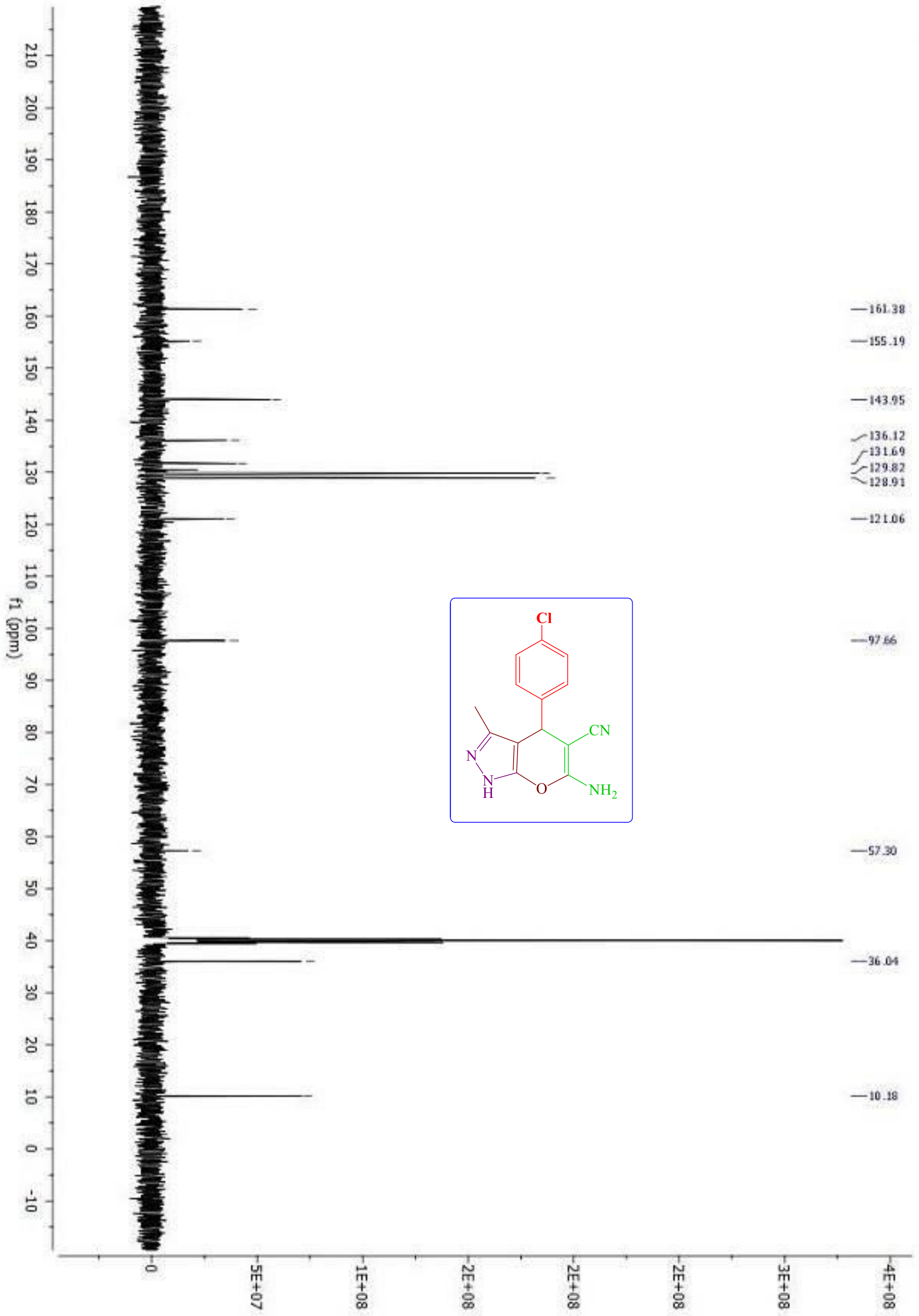

Figure S2. ^{13}C NMR spectrum of compound (**5b**)

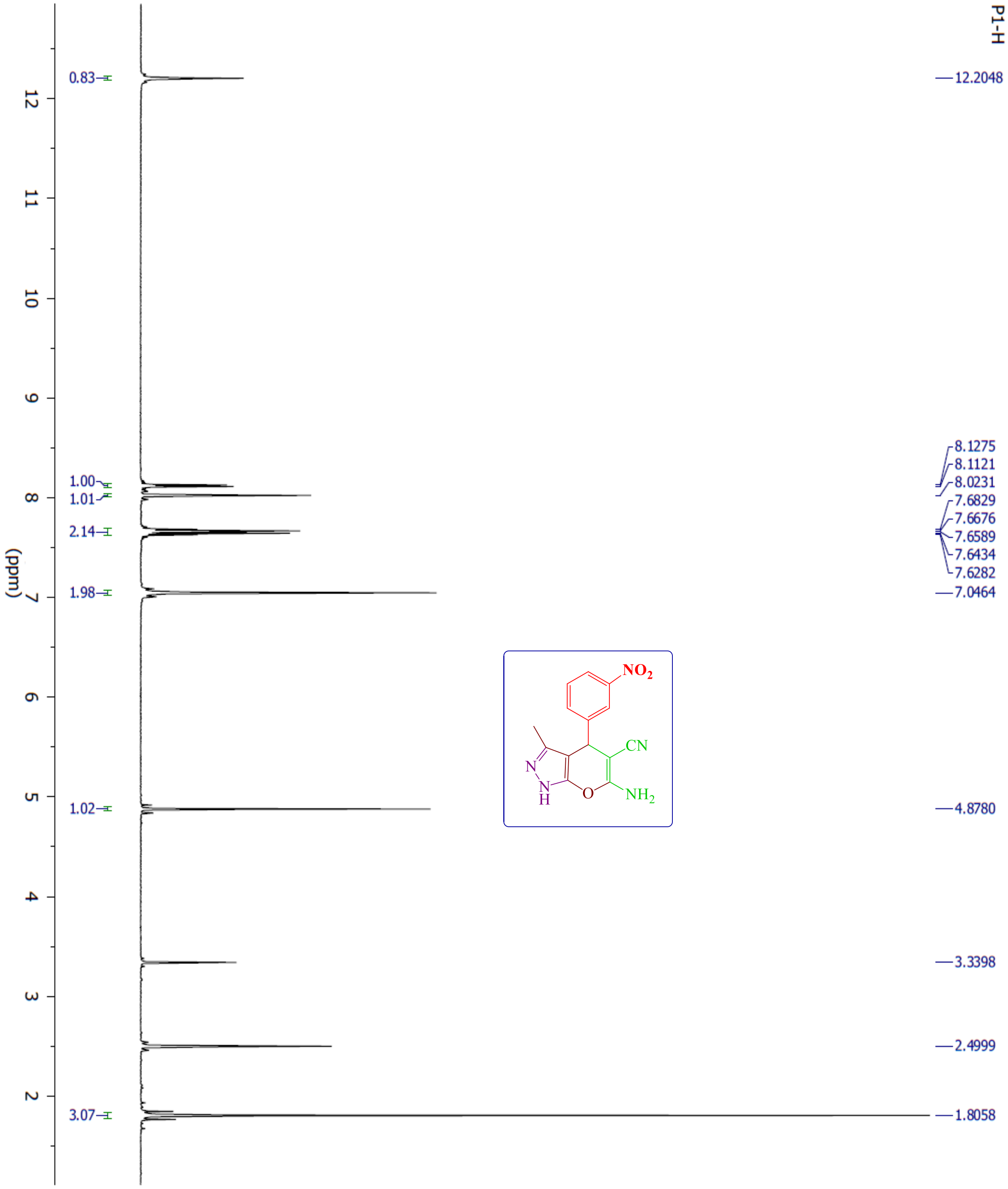

Figure S3. ^{13}H NMR spectrum of compound (**5e**)

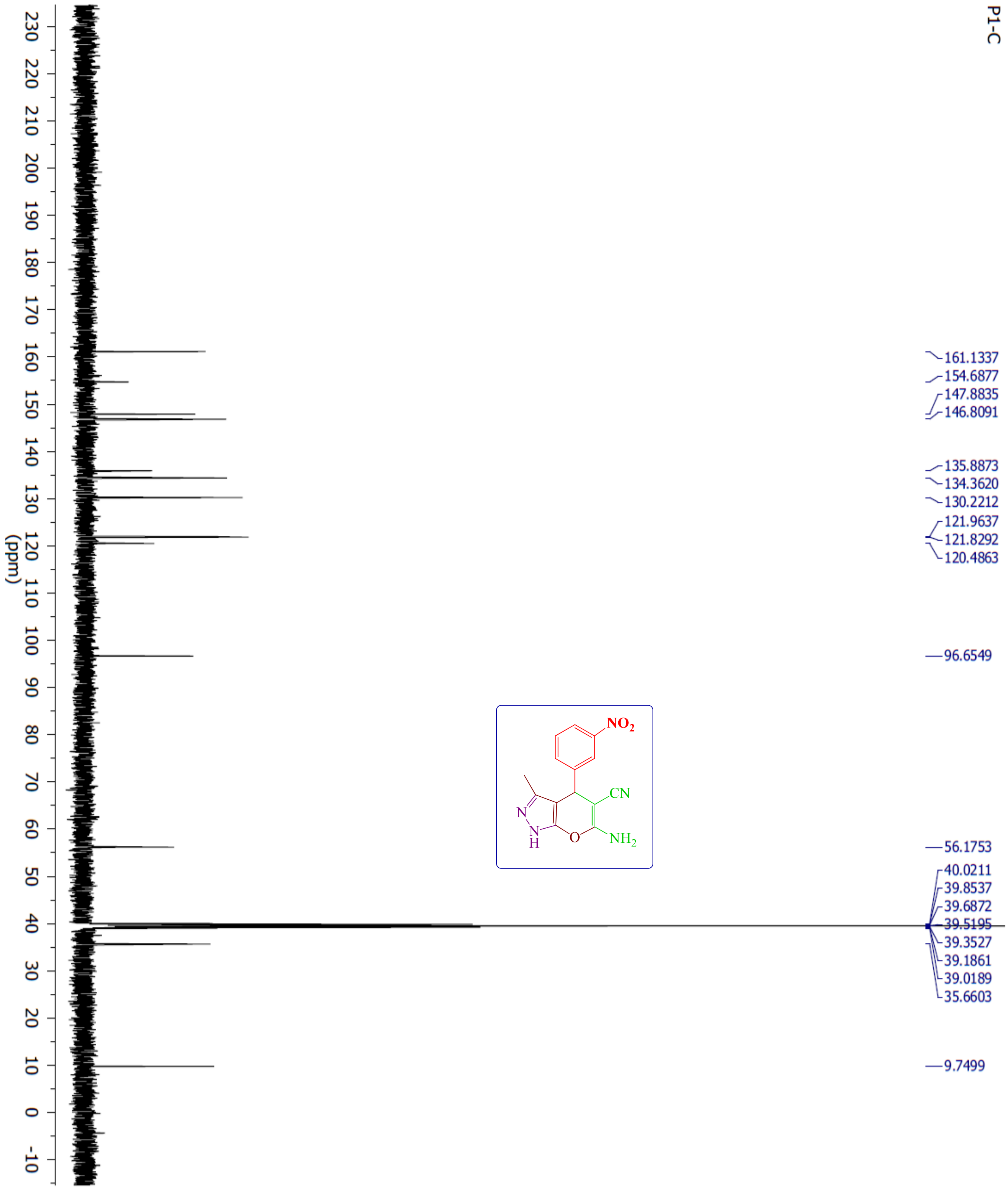

Figure S4. ^{13}C NMR spectrum of compound (**5e**)

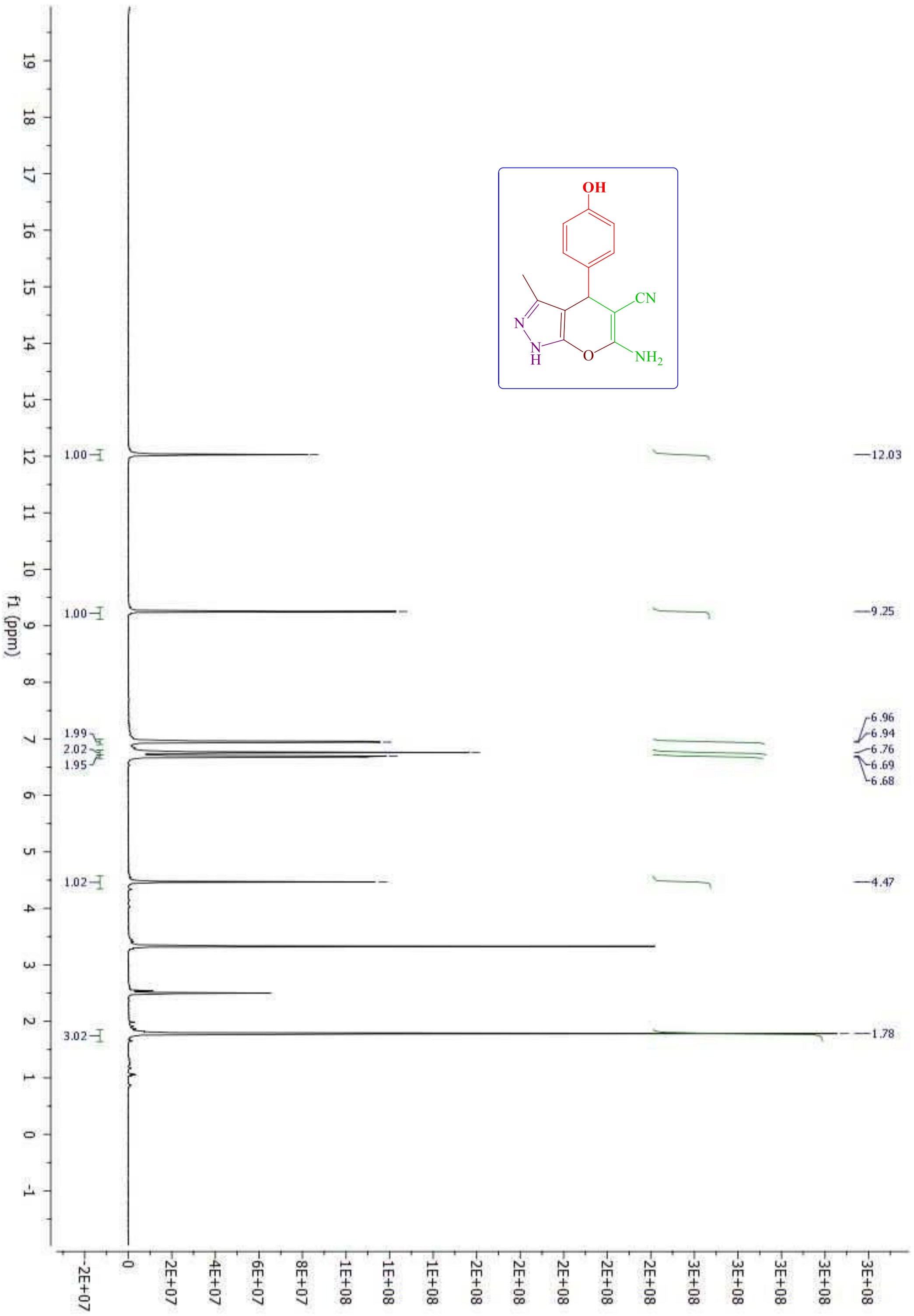

Figure S5. ^{13}H NMR spectrum of compound (**5j**)

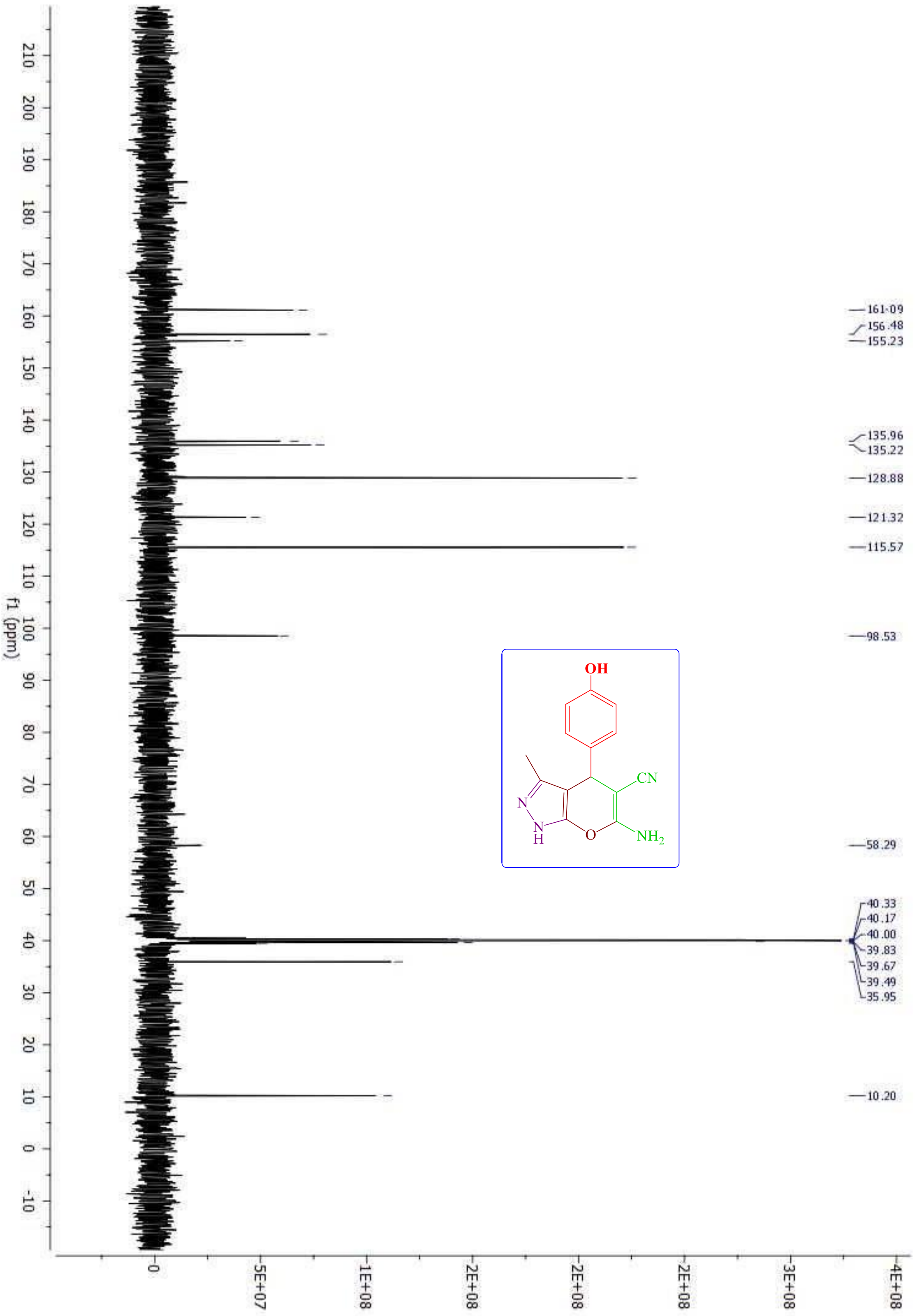

Figure S6. ^{13}C NMR spectrum of compound (**5j**)
